# An Overview on *Citrus aurantium* L.: Its Functions as Food Ingredient and Therapeutic Agent

**DOI:** 10.1155/2018/7864269

**Published:** 2018-05-02

**Authors:** Ipek Suntar, Haroon Khan, Seema Patel, Rita Celano, Luca Rastrelli

**Affiliations:** ^1^Department of Pharmacognosy, Faculty of Pharmacy, Gazi University, Etiler, 06330 Ankara, Turkey; ^2^Department of Pharmacy, Abdul Wali Khan University, Mardan 23200, Pakistan; ^3^Bioinformatics and Medical Informatics Research Center, San Diego State University, San Diego, CA 92182, USA; ^4^Dipartimento di Farmacia, University of Salerno, 84084 Fisciano, Italy

## Abstract

*Citrus aurantium* L. (Rutaceae), commonly known as bitter orange, possesses multiple therapeutic potentials. These biological credentials include anticancer, antianxiety, antiobesity, antibacterial, antioxidant, pesticidal, and antidiabetic activities. The essential oil of *C. aurantium* was reported to display marked pharmacological effects and great variation in chemical composition depending on growing locations but mostly contained limonene, linalool, and *β*-myrcene. Phytochemically, *C. aurantium* is rich in *p*-synephrine, an alkaloid, and many health-giving secondary metabolites such as flavonoids. Animal studies have demonstrated a low affinity of *p*-synephrine for adrenergic receptors and an even lower affinity in human models. The present review focuses on the different biological activities of the *C. aurantium* in animal and human models in the form of extract and its pure secondary metabolites. Finally, it is concluded that both the extract and isolated compounds have no unwanted effects in human at therapeutic doses and, therefore, can confidently be used in various dietary formulations.

## 1. Introduction

*Citrus aurantium* L. (Rutaceae), commonly known as bitter orange, is usually utilized as a flavoring and acidifying agent for food [1]. Besides the essential oil and its components [2, 3], the fruits of *C. aurantium* are sources of flavonoid-type compounds with diverse biological effects [4–6]. Additionally, it was reported that flavonoid glycosides were isolated from the plant [7] and the biogenic amine and flavanone contents have been determined [8, 9]. Due to the abundance of health-giving secondary metabolites, *C. aurantium* is also used for the treatment of several ailments such as anxiety [10], lung and prostate cancers [11], and gastrointestinal disorders and obesity [2, 12]. Due to the prohibition of *Ephedra sinica* Stapf. in Farw.-containing weight loss products in the market, *C. aurantium* has found an important place as a preferable agent to replace ephedra, as it contains *p*-synephrine, a phenylethanolamine type alkaloid, which is chemically similar to adrenergic agents, as appetite suppressants [12–14]. Recently, several scientific studies investigating the potential effects of various parts (including flowers, fruits, and essential oils) of *C. aurantium* have been conducted [15–17].

In the last years, phytopharmaceuticals have shown an outstanding role in new drug discovery [18–20]. Both in the crude form as well as pure chemical entities, a large population around the globe are getting therapeutic benefits from them [21–23]. In this review article, we have aimed to overview the bioactivity studies performed on *C. aurantium* revealing its therapeutic potential in the light of isolated molecules and/or essential oils.

## 2. Chemistry of *Citrus aurantium*

The chemical composition of *C. aurantium* is responsible for health-promoting effects. The chemical composition includes vitamins, minerals, phenolic compounds, and terpenoids [21–23]. Among the diverse chemical components in *C. aurantium*, flavonoids belonging to phenolics have been recognized as important due to their physiological and pharmacological role and their health benefits [21–23].

**Scheme 1:**
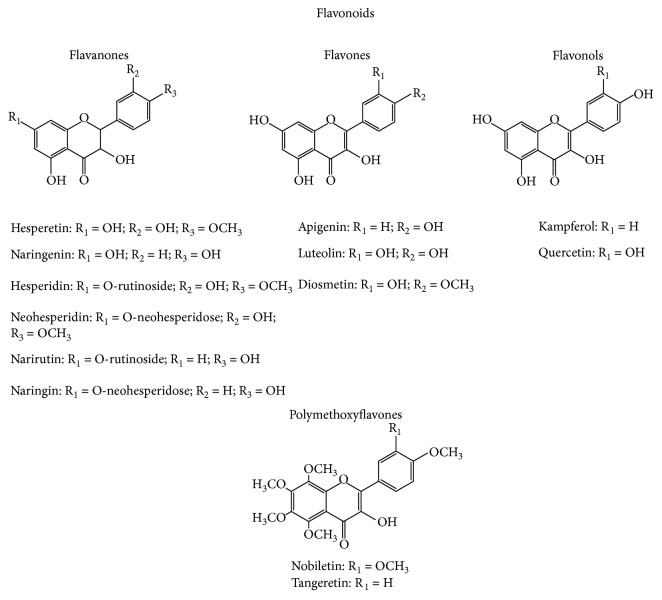
Chemical structures of major flavonoids contained in *C. aurantium.*

The flavonoids contained in *C. aurantium* can be divided into four groups, including flavones, flavanones, flavonols, and anthocyanins (only in blood oranges) (Scheme 1)[21–23].

Flavonoids are mainly present in *Citrus* fruits as glycosyl derivatives. Aglycones are mainly present in specific parts of the fruit as peel and seeds, owing to their lipophilic nature and consequently their low solubility in water [21–23]. For glycoside forms, O-glycosides, C-glycosides, rutinosides, glucosides, and neohesperidosides are common [21–23]. The presence of a relatively large number of glycosylated flavonoids in *Citrus* is a result of many different combinations possible between polyhydroxylated aglycones and a limited number of mono- and disaccharides [24].

Flavanones are the main flavonoids contained in *C. aurantium* [25]. The most abundant detected free flavanones are hesperetin (4′-methoxy-3′,5,7-trihydroxyflavanone) and naringenin (4′,5,7-trihydroxyflavanone) [26]. Hesperetin and naringenin present a common skeleton: two hydroxyl group in positions C-5 and C-7, respectively, and they can be found as aglycone and/or as glycosides [25]. The most widely distributed glycosides of hesperetin are hesperidin and neohesperidin, which are conjugates with rutinose and neohesperidose, respectively, while, for naringenin, the most abundant glycosyl derivatives are naringin (naringenin-7-neohesperidoside) and narirutin (naringenin-7-rutinoside) [25].

The flavones in aglycon or/and glycosidic form are the second major group of flavonoids in *C. aurantium*. The most commonly detected free flavones are apigenin, luteolin, and diosmetin. O-Glycosides and C-glycosides are the two main forms of flavone glycosides, and the most common linked sugar moieties include glucose, rutinose, and neohesperidose [26].

In *C. aurantium,* the flavones may be present also in the methoxylated form, in which all or almost all hydroxyls are capped by methylation, as nobiletin and tangeretin [27]. Furthermore, the *C. aurantium* may contain low amounts of flavonols, as kaempferol and quercetin, mainly in glycosidic form.

**Scheme 2:**
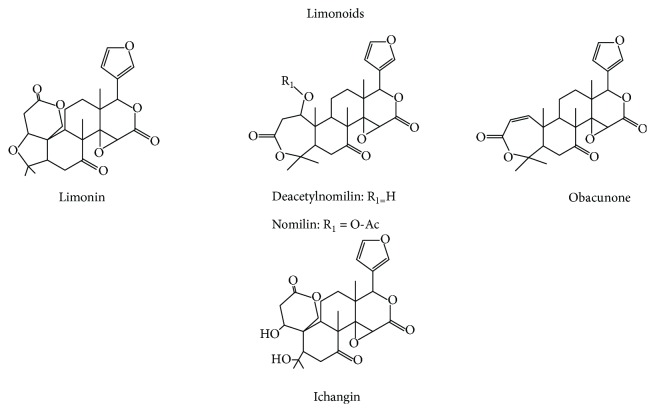
Chemical structures of major Limonoids contained in *C. aurantium.*

The second class of secondary metabolites found in *C. aurantium* are the limonoids (Scheme 2). The latter were considered as oxygenated triterpenoids as they contain relatively high numbers of oxygen atoms (7–11) in their structures. All components have a furan ring attached to the D-ring at C-17. Limonoids occur both in glucosidic and aglyconic form. Limonoid aglycones are water-insoluble and responsible for a bitter taste of the *Citrus* fruits, while limonoid glucosides are water-soluble and tasteless [28]. Among limonoids, the most important one is limonin, known as *Citrus* constituent since 1841. Limonoid glucosides are more abundant in juices and pulps, such as limonin glucoside, nomilin glucoside, obacunone glucoside, nomilinic acid glucoside, and deacetylnomilinic acid glucoside, because they are water-soluble, while limonoid aglycones such as limonin, nomilin, obacunone, ichangin, and deacetyl nomilin are water-insoluble and are present mainly in seeds and peels [29, 30].

Another class of compounds contained in *C. aurantium* are phenylethylamine alkaloids with *p*-synephrine being the most abundant. This compound has a hydroxyl group in the para position on the benzene ring and has some structural similarity to ephedrine. The peel of unripe fruits is the part of the plant which has the highest level of *p*-synephrine [31].

Zarrad and coworkers [32] explored the chemical composition of Tunisian *C. aurantium* fruits using gas chromatography (GC) and gas chromatography-mass spectroscopy (GC-MS). 25 compounds were identified in 97.696% exploration, but the most prominent one was limonene with 85.52%, followed by linalool and *β*-myrcene with 3.365% and 1.628%, respectively. The remaining compounds were in traces. Earlier, Villafane et al. [33] reported the presence of D-limonene and nootkatone in the essential oil of the plant. Similarly, Sanei-Dehkordi et al. [34] identified 21 compounds in peel essential oil of the plant. Of the total explored 98.62% composition, the quantity of limonene was 94.81% while the LC_50_ was 31.20 ppm against *Anopheles stephensi.* However, the concentration of limonene was only 0.5–2.5% in *C. aurantium* from Algeria and linalool and *α*-terpineol were 18.6% and 15.1%, respectively [35]. Thus, overall considerable variation was observed in the concentration of these major components in *C. aurantium* grown in different parts of the world [36–41]. Carvalho-Freitas and Costa observed strong anxiolytic and sedative-like effects of the essential oil derived from *C. aurantium* [42].

## 3. Pharmacological Profile

### 3.1. Cytotoxic and Anticancer Effects

The anticancer activity of the plant has been reported in literature [26, 43]. Cytotoxic properties of the polysaccharides obtained from *C. aurantium* were examined. Cytotoxic activity on human breast cancer cells (MCF-7) and lung cancer cells (HCC827) and immune-enhancement effect were examined. The results indicated that *C. aurantium* var. *amara* polysaccharides displayed good immune-enhancement activity by stimulating the production of tumor necrosis factor-*α* (TNF-*α*) and interleukin-6 (IL-6) in RAW264.7 cells and by promoting the mRNA expression levels of inducible nitric oxide synthase (iNOS), TNF-*α*, interleukin-1*β* (IL-1*β*), and IL-6. Moreover, phosphorylated extracellular signal-regulated kinase (ERK), phosphorylated c-Jun N-terminal kinase (JNK), phosphorylated p38, and phosphorylated p65 were significantly enhanced in *C. aurantium* var *amara* polysaccharides-treated RAW264.7 cells [24, 44]. Isolimonic acid and ichanexic acid from ethyl acetate extract of *C. aurantium* were isolated by using chromatographic methods. The compounds for their inhibitory action on human colon cancer cells (HT-29) proliferation, apoptosis, and on noncancerous (COS-1 fibroblast) cells were investigated. The compounds displayed an increment in the cell counts in G2/M stage, demonstrating a potential effect in the cell-cycle arrest [25].

Protective activity of the extract obtained from the peels of *C. aurantium* against apoptosis in cholestatic liver fibrosis-induced mice was investigated. As part of the experiment, cholestatic liver injury was induced by bile duct ligation and the mice were treated with *C. aurantium* peel extract. According to the histopathological analysis, the administration of *C. aurantium* peel extract significantly decreased liver fibrosis. Biochemical analysis revealed that the extract reduced the concentrations of alanine transaminase (ALT), aspartate transaminase (AST), gamma-glutamyl transferase, total bilirubin, nitric oxide, and thiobarbituric acid reactive substances (TBARS), suggesting *C. aurantium* can efficiently regulate bile duct ligation-induced liver injury by displaying antioxidant, anti-inflammatory, and antiapoptotic effects [27].

**Figure 1:**
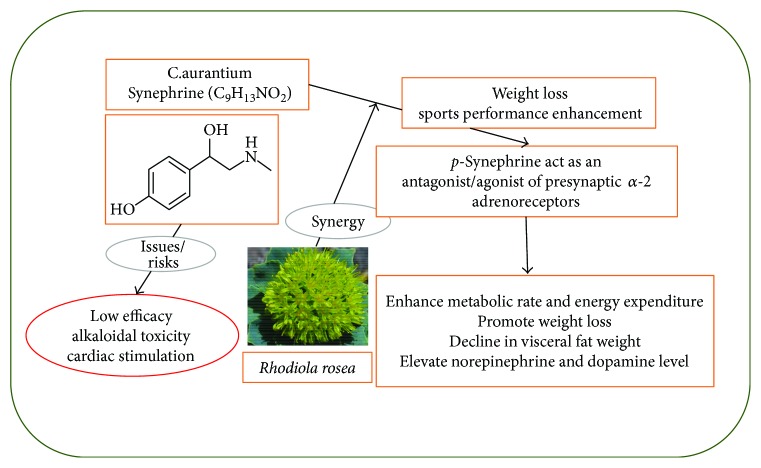
Cytotoxic effects and mechanisms of *C. aurantium* extracts.

*C. aurantium* was shown to possess antimetastasis activities in *in vitro* assays. A study by Park et al. evaluated the antimetastatic effect of flavonoids, isolated from Korean *C. aurantium* in mice models. Flavonoids were shown to prevent cancer cell infiltration and localization to the lungs. Moreover, through the regulation of caspase-3 and phospho-p53, the flavonoids induced cancer cell apoptosis. *In vitro* tests demonstrated that flavonoids inhibited A549 cells metastasis and induced apoptosis and downregulation of the Ddx3x and ANP43B proteins, supporting the use of *C. aurantium* flavonoids in human lung cancer treatment. According to the bioactivity assays conducted on the major compounds of *C. aurantium* var. *amara*, namely, 5-hydroxy-6,7,30,40-tetramethoxyflavone (HTF) and limonexic acid (LA), both compounds showed remarkable antioxidant effects and notable inhibitory action on the B16 and SMCC-7721 cell lines, at a concentration between 6.25 and 50 *μ*g/mL and between 12.5 and 200 *μ*g/mL, respectively [28].  Figure 1 illustrates the cytotoxic effects and mechanisms of *C. aurantium* extracts.

Miyazawa et al. [45] isolated three polymethoxy flavonoid-type compounds, namely, tetra-*O*-methylscutellarein, sinensetin, and nobiletin from *C. aurantium*. These compounds caused marked downstream regulation of a variety of gene expressions and thus showed strong antimutagenic activity. Wang and coworkers [46] summarized the anticancer effects of polymethoxyflavones with detailed molecular mechanisms. The various amines and flavonoids have been quantitatively analyzed in *C. aurantium* using liquid chromatography [47, 48]. Hesperidin has been isolated from *C. aurantium* [49]. It displayed a significant apoptotic effect against liver cell lines, HepG2 cells, by mediating through the upstream regulation of mitogen-activated protein kinase ERK1/2 [50].

### 3.2. Anxiolytic and Sedative effects

The anxiolytic and sedative effects of *C. aurantium* by *in vivo* light-dark box and the marble-burying assays were evaluated*. C. aurantium* essential oil enhanced the period the mice spent in the light chamber, as well as the number of transitions between the two compartments in the light-dark box test [29]. Moreover, single and repeated treatments with the essential oil were reported to suppress marble-burying behavior [10]. The anxiolytic activity of the blossom of *C. aurantium* was also evaluated clinically on 60 patients undergoing minor surgical operation with no organic pathology. Two hours before the anesthesia induction, two groups consisting of 30 patients were administered at 1 mL/kg of either *C. aurantium* distillate or saline solution. Anxiety was assessed by using the Spielberger state-trait anxiety inventory, the Amsterdamd preoperative anxiety, and information scale. The outcome of this study revealed that *C. aurantium* could be active by reducing preoperative anxiety before minor operation [30].

In traditional Chinese medicine (TCM), the combination of the aqueous extracts of the fruits of *Gardenia jasminoides* Ellis, *C. aurantium*, and the bark of *Magnolia officinalis* Rehd. et Wils. is called Zhi-Zi-Hou-Po (ZZHPD) and has been used to treat depression-like symptoms. Xing et al. [51] explored *in vivo* antidepressant activity of ZZHPD in rats, by using coat state test, sucrose preference test, forced swimming test, and open-field test. The effects of ZZHPD on hypothalamic-pituitary-adrenal (HPA) axis were determined by measuring the hormone levels. The results showed that ZZHPD improved depressive behaviors and normalized the adrenocorticotropic hormone (ACTH) and corticosterone levels, by restoring the function of HPA axis, and increasing brain-derived neurotrophic factor expression in hippocampus and promoting hippocampal neurogenesis [51]. ACTH/corticotropinis a peptidic hormone secreted from the anterior pituitary, which regulates glucocorticoid cortisol production from the adrenals [52]. Stress increases ACTH release, which induces the stress hormone cortisol, together they cause numerous diseases [53].

Letie et al. [54] conducted another study *in vivo*, where rat models were exposed to the essential oil of *C. aurantium* at 1.0%, 2.5%, and 5.0% concentrations by inhalation, in acrylic boxes before employing elevated plus maze and open-field assays. *C. aurantium* essential oil increased the time of the animals in the open arms of the elevated plus maze test and the time of active social interaction in the open field [54].

Wolfenbuttel et al. [55] investigated the influence of *C. aurantium* essential oil on melatonin and corticosterone in mice after inhalation for 30 min. Melatonin provides protective effects on neuronal cells and acts as an antidepressant by restoration of corticosterone levels. After treatment, the hormone levels did not present variation, whereas behavioral tests showed that the inhalation of 10% essential oil causes an anxiolytic-like and sedative effect. From these data, *Citrus* essential oil represents a valuable tool for the treatment of the anxiety disturbs, apparently without interference with melatonin and corticosterone physiological levels [55].

### 3.3. Antidiabetic Effects

Several reports have been cited in literature regarding the antidiabetic effects of *C. aurantium* [56–58]. An *in vivo* study was performed to evaluate the antidiabetic effect and to reveal the toxicity profile of the aqueous extract of the fruits of *C. aurantium* and the leaves of *Rauwolfia vomitoria*. In this study, NMRI lean mice (6-week-old) and C57BL/6J lean mice (6- or 11-week-old) were used. A single dose, which corresponds to seventy-fold of a human daily dose, was detected to be nontoxic to the animals. A significant weight loss was determined when the dosage, which corresponds to tenfold of a human daily dose, was administered to C57BL/KsBom-*db* (*db*/*db*) genetic diabetic mice for 6 weeks. These mice were maintained on the carbohydrate-deficient diet during the treatment period. The food intake was not significantly different from the control group animals; however, the serum triglyceride levels of the treated animals were significantly higher suggesting the lipid mobilization from internal stores. The fatty acid levels of the eyes of the treated mice remarkably reduced along with stearoyl-CoA desaturase activity [59]. A possible effect of the extract obtained from *C. aurantium* and *p*-synephrine on liver metabolism was evaluated. In order to measure catabolic and anabolic pathways, an isolated perfused rat liver was used. Both the extract and the compound were found to enhance glycolysis, glycogenolysis, oxygen uptake, and perfusion pressure. *p*-Synephrine increased the glucose output at 200 *μ*M concentration. *C. aurantium* extract enhanced gluconeogenesis at low concentrations, however, inhibited at high concentrations. The effects of *C. aurantium* extract on liver metabolism was found to be similar to those of adrenergic agents, and *p*-synephrine could be responsible from the activity [14].

### 3.4. Antiobesity Effects

Besides its scientifically proven antimicrobial, antioxidant, cytotoxic, anxiolytic, and antidiabetic effects, *C. aurantium* extract has been commonly utilized for the weight loss and as sports performance enhancer, in dietary supplements [60–62]. Therefore, the use of *C. aurantium* extract and its constituent *p*-synephrine (C_9_H_13_NO_2_), for the treatment of obesity in 360 subjects, was reviewed. More than 50% of the subjects involved in these clinical studies were overweight, and approximately two-thirds of them consumed caffeine (132–528 mg/day) and *p*-synephrine (10–53 mg/day). Approximately 44% of the subjects used a *C. aurantium*/*p*-synephrine product, while the remaining consumed a combination product containing multiple ingredients with *p*-synephrine. The results showed that *C. aurantium* extract alone or in combination with other ingredients did not cause significant adverse effects including an increase in heart rate or blood pressure or change in electrocardiographic data, serum chemistry, blood cell counts, or urinalysis. *p-*Synephrine, alone or in combination products, was demonstrated to enhance metabolic rate and energy expenditure and to promote weight loss when given for six to 12 weeks [63].

**Figure 2:**
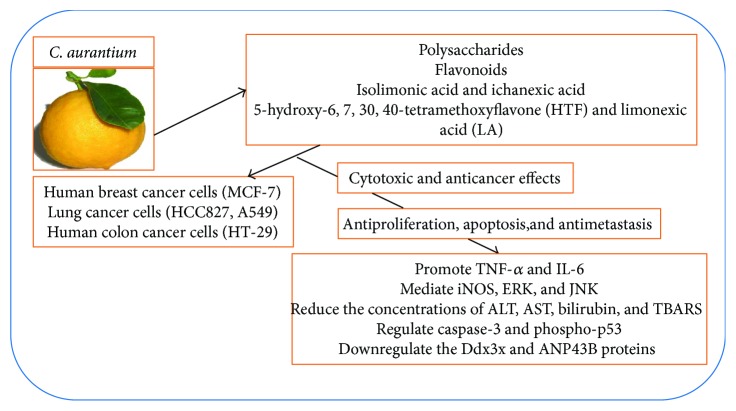
Pharmacological effects of *C. aurantium* extracts.

The study by Verpeut et al. [64] investigated the effect of the combination of *C. aurantium* (standardized to 6% *p*-synephrine) and *Rhodiola rosea* L. (golden root) (standardized to 3% rosavins and 1% salidroside) on diet-induced obesity in Sprague-Dawley rats. Acute administration of *C. aurantium* (1–10 mg/kg) or *R. rosea* (2–20 mg/kg) alone did not decrease food intake in normal weight animals; however, the combination of *C. aurantium* (5.6 mg/kg) and *R. rosea* (20 mg/kg) provided a 10.5% feeding suppression. On the other hand, 10 days of treatment with *C. aurantium* (5.6 mg/kg) or *R. rosea* (20 mg/kg) alone, or in combination, to the animals fed on a high-fat diet (60% fat) during the 13-week period led to a 30% decline in visceral fat weight, compared with other treatments. Coadministration of *C. aurantium* and *R. rosea* also resulted in an elevation in hypothalamic norepinephrine and frontal cortex dopamine, indicating the beneficial role of *C. aurantium* and *R. rosea* in the treatment of obesity [64].  Figure 2 illustrates the various pharmacological effects of *C. aurantium* extracts.

### 3.5. Effects on the Cardiovascular System

The cardiovascular toxicity of *C. aurantium* extracts with different concentrations of protoalkaloid *p*-synephrine (4 and 6%) was reported by Calapai et al. in 1999 in rats. Authors showed an antiobesity effect by the administration of *C. aurantium* but also possible cardiovascular toxicity. The cardiovascular effects have not been confirmed by studies using much higher doses of *p*-synephrine [65].

Another study was carried out to evaluate the cardiovascular effects of different doses of *C. aurantium* and pure *p*-synephrine in rats. *C. aurantium* extract and pure *p*-synephrine enhanced the heart rate and blood pressure. Higher activities were obtained with *C. aurantium* extract than *p*-synephrine, suggesting that other compounds in the extract can alter physiological parameters [13]. The effect of *p*-synephrine on heart rate and blood pressure in female Sprague-Dawley rats was assessed. During 28 days, two types of extracts, one of which contained 6% and other 95% *p*-synephrine, were administered daily by gavage at 10 or 50 mg/kg, doses for a 60 kg human equal to 600 and 3000 mg/kg. The outcome of the study showed that both *p*-synephrine and *C. aurantium* extract resulted in clinically insignificant increases in heart rate and blood pressure at doses many times greater than used in humans. *p*-Synephrine and bitter orange extract exhibited little or no effect on the cardiovascular effects of caffeine [13].

Safety of *p*-synephrine was investigated by Ratamess et al. [66] on humans, animals, and *in vitro*. Authors reported over 30 human studies indicating that the cardiovascular effects of *p*-synephrine and bitter orange extracts are clinically insignificant. *p*-Synephrine showed a greater ability to bind adrenergic receptors in rodents than in humans, and the data in literature on its effects on animals cannot be indicative for men at commonly used doses.

This review concluded that *C. aurantium* extract and *p*-synephrine are safe for use in dietary supplements and foods at commonly used doses. Also, other authors reported similar conclusions [67, 68]. While *p*-synephrine alone seems to have low toxicity, when it is formulated in combination with other ingredients such as caffeine (*Paullinia cupana*, *Cola nitida*, *Cola acuminata*, and *Camellia sinensis*), salicin (*Salix* sp.), and ephedrine (ma huang, *Ephedra sinica*, and *Ephedra* sp.) in weight loss products, the mixture could induce some cardiovascular effects. However, the study did not demonstrate that *p*-synephrine contributed to cardiovascular effects.

Schmitt et al. [69] reported clear signs of toxicity of this mixture in mice of both sexes as reduction in locomotor activity, ptosis, seizures, salivation, agitation, piloerection, and deaths after acute oral administration of 300, 350, and 400 mg/kg total of *p*-synephrine, ephedrine, salicin, plus caffeine in a 10 : 4 : 6 : 80 *w*/*w* ratio.

The volatile oil obtained from the flowers of *C. aurantium* var. *amara*, namely, neroli oil, is used to reduce heart rate and palpitations, to encourage sleep, and to soothe the digestive tract. Kang et al. [15] investigated the activity mechanism of neroli in mouse aorta. Neroli was found to exert vasodilator activity in mice precontracted with prostaglandin (PG)-F2*α*. Nevertheless, the relaxation was reduced in the endothelium-denuded ring or preincubation with the nitric oxide synthase inhibitor by neroli treatment. Moreover, the relaxation induced by neroli was partially reversed by soluble guanylyl cyclase inhibitor. Neroli also inhibited extracellular Ca^2+^-dependent and depolarization-induced contraction in a dose-dependent manner. Nonselective cation channel blocker, Ni^2+^, decreased neroli-induced relaxation. On the other hand, a K^+^ channel blocker, tetraethylammonium chloride, did not alter the relaxation. In order to prevent Ca^2+^ influx through the smooth muscle voltage-gated Ca^2+^ channels, verapamil was added. In this case, the ryanodine (a class of intracellular calcium channels) receptor inhibitor reduced neroli-induced relaxation. All these data indicated that neroli-induced relaxation might be partly mediated by the nitric oxide-soluble guanylyl cyclase, and ryanodine receptor signaling pathway [15].

### 3.6. Effect on Microorganisms

Antimicrobial activity of *C. aurantium* was investigated by using several *in vitro* assays [70–72]. For instance, Karabıyıklı et al. [1] explored the antimicrobial action of *C. aurantium* juice against *Salmonella enterica* Typhimurium and *Listeria monocytogenes.* For this purpose, both neutralized and unneutralized juice samples with various concentrations were tested for the inoculation of the microorganisms at 4°C and 37°C temperatures, during a period of seven days. The results showed that *Salmonella enterica* Typhimurium and *Listeria monocytogenes* not only survived but also grown for two days in neutralized juice at 37°C. On the other hand, on day 7, none of them survived. The results also revealed that *L. monocytogenes* was less resistant than *S. enterica* Typhimurium. The low pH of *C. aurantium* juice was suggested to be responsible for its antimicrobial potential along with the duration of the incubation period as well as the temperature [1]. In another research, the antimicrobial potential of *C. aurantium* was investigated, and high antimicrobial activity was recorded against *Bacillus subtilis* and *Staphylococcus aureus* (among 12 microorganisms tested), with the minimum inhibition concentration (MIC) values of 2.7 mg/mL and 4.8 mg/mL. Moderate effects were detected against *Saccharomyces cerevisiae* and *Mucor ramannianus* with the values of 9.2 mg/mL and 5 mg/mL, respectively. In the same study, radical scavenging and antibacterial effects of the essential oil obtained from the leaves of *C. aurantium* were evaluated. Weak antioxidant effect was detected against 1,1-diphenyl-2-picrylhydrazyl (DPPH) and 2,2′-azinobis-3-ethylbenzothiazoline-6-sulphonate (ABTS) radicals [73].

### 3.7. Pesticidal Effects

Previous *in vitro* investigations on the essential oil of *C. aurantium* peels and its isolated component limonene have demonstrated their pest fumigant activity against *Bemisia tabaci* (silverleaf whitefly). In 24 h exposure, insect mortality between 41.00 and 47.67% was determined at 2.5 and 20.0 *μ*L/L air concentrations. In the same study, the authors conducted another assay, investigating the anticholinesterase effect of the oil itself, as well as that of the pure compound. Both exerted anticholinesterase effect with the IC_50_ values of 2.94 mM and 3.54 mM, respectively [74–76]. Other studies investigated the larvicidal effect of the essential oils of *C. aurantium* against *Anopheles labranchiae*. After 24 h, the mortality counts were carried out, and LC_50_ and LC_90_ values were determined. The outcome of this study demonstrated that the essential oils exerted significant larvicidal potential. *C. aurantium* essential oil was found to be the most active by the LC_50_ and LC_90_ values of 22.64 mg/L and 83.77 mg/L, respectively [13, 77]. The essential oil extract from the fresh peeled ripe fruit of *Citrus aurantium* showed also good larvicidal effect against mosquito vector *Anopheles stephensi* (LC_50_ values, 31.20 ppm) The main constituent of the leaf oil was limonene (94.81).

### 3.8. Antioxidant Effects

DPPH, ABTS, and ferric-reducing antioxidant power assays were used for the determination of the antioxidant potential of the macerate of the albedo layers of *C. aurantium* fruits obtained by protopectinase-SE (produced by *Geotrichum klebahnii* and hydrolyzed selectively the intercellular protopectin of plant tissues). Moreover, the levels of total phenols, reducing sugars, vitamin C, total flavonones, naringin, and galacturonic acid and total acidity were detected. Antioxidant activity, vitamin C, and total flavonone levels were found to be the highest with the greatest degree of tissue maceration [78–81]. *In vivo* and *in vitro* antioxidant activities of polysaccharide fractions from *C. aurantium* were evaluated. The most active fraction was subjected to ion exchange and gel-filtration chromatography to obtain four purified polysaccharides. Upon the evaluation of their antioxidant effect, it was found that *C. aurantium* can be utilized as an antioxidant in the food and medical industries [82, 83].

In a study by Lagha-Benamrouche et al. [84], the antioxidant potentials of the peels and leaves of seven orange varieties obtained from Algeria were investigated by linoleic acid and *β*-carotene oxidation assays. The presence of phenolic compounds was also investigated. *C. aurantium* cv. Bigarade was found to have the highest phenol level. The antioxidant activity assay was in accordance with the phytochemical findings. *C. aurantium* displayed the highest action on slowing down the rate of linoleic acid and *β*-carotene oxidation [84, 85]. An antioxidant study was performed on the *C. aurantium* fruits on different ripening stages. According to the outcome obtained from DPPH free radical-scavenging and *β*-carotene/linoleic acid systems, the antioxidant activity was found to be varied related to the amount of the phenolic components [86]. Another antioxidant activity study revealed that during dehydration, different air-drying temperature affected the antioxidant effect of the *C. aurantium* by-products, including peel and pulp remaining after juice extraction [79]. *C. aurantium* peel and juice were suggested as a new potential source of natural antioxidants; although, they were found to be less effective than butylated hydroxytoluene (BHT), butylated hydroxyanisole (BHA), and ascorbic acid, used as antioxidant standards [87, 88]. Anti-inflammatory activity of the flavonoid-type compounds of Korean *C. aurantium*, namely, nobiletin, naringin, and hesperidin was investigated. Inhibition of proinflammatory mediators by blocking nuclear factor-kappa B (NF-ƘB) and mitogen-activated protein kinase (MAPK) signaling in lipopolysaccharide- (LPS-) stimulated RAW 264.7 macrophages was assessed [89]. The flavonoids were found to have the capacity to suppress the mRNA and protein expression of COX-2 and iNOS, by clarifying their anti-inflammatory action [4]. A polymethoxy flavonoid-rich *C. aurantium* extract was shown to have a protective effect on alcohol-induced liver injury in an animal study via AMPK and Nrf2-related signal regulation [90].

### 3.9. Antiulcer Effects

In order to assess the effect of *C. aurantium* essential oil and its main compound limonene on gastric mucosa, Moraes et al. [2] conducted a study *in vivo*. The essential oil and its compound limonene were found to possess protective activity in the gastrointestinal system against lesions, which were induced by ethanol and nonsteroidal anti-inflammatory drugs in rats, at doses of 250 mg/kg and 245 mg/kg, respectively. The essential oil and limonene increased the production of gastric mucus. The findings revealed that *C. aurantium* essential oil and its main compound limonene can be used as a promising target for the development of a novel gastroprotective drug [2]. In another study, the gastroprotective effect of *β*-myrcene, a monoterpene-type compound of *C. aurantium*, was evaluated. Experimental models of ulcer, induced by ethanol, NSAID stress, *Helicobacter pylori*, ischemia-reperfusion injury (I/R), and cysteamine (a drug used to treat cystinosis) was used to assess the ameliorative activity. *β*-Myrcene was administered at dose of 7.5 mg/kg. The results showed a potential role for *β*-myrcene against peptic ulcer disease. *β*-Myrcene contributed to the maintenance of integrity of the gastric mucosa with a significant decrease of ulcerative lesions, attenuating lipid peroxidative damage and preventing depletion of GSH, GR, and GPx [91].

Polo et al. confirmed the healing properties of *C. aurantium* essential oil on gastric ulcers after treatment in middle-aged Wistar rats. They showed a significant reduction of the lesion area (76%) within the gastric mucosa that appears regenerated (59%) when compared to the negative control group [92].

On the other hand, Hamdan et al. assessed the effects of hesperidin and neohesperidin, important flavonoid-type components of *C. aurantium*. For this purpose, indomethacin-induced ulcer models of rats were used. Omeprazole was administered as a reference standard for the comparison. The parameters analyzed in the present study were ulcer index, gastric cyclooxygenase-2 (COX-2) gene expression, TNF-*α*, MDA, and GSH levels. Histopathalogical analysis was also performed. The findings of the study revealed that hesperidin and neohesperidin notably aggravated indomethacin-induced gastric damage as evidenced by increased ulcer index and histopathological alteration [93]. Recently, hesperidin showed strong inhibition against ovarian cancer cell viability and caused apoptosis mediated through endoplasmic reticulum stress signaling pathways [94]. Hafidh et al. [95] observed the potent anticancer effects of limonene in hepatocellular carcinoma and HepG2, and the cell line caused the modulation of cancer-inducing genes. These data confirmed the antioxidant and prooxidant behavior of flavonoids showing the potential benefits and adverse effects of these opposing events.

## 4. Adverse Effects

Because of the possible adverse effects of *C. aurantium* in the cardiovascular system, the use of *C. aurantium*-containing products has been questioned mainly due to the *p*-synephrine content [96]. As a fat decreasing agent, it was used in traditional medications, but it has been banned by the National Collegiate Athletic Association (NCAA), due to its risks such as cardiovascular hazards and alkaloid toxicity [97]. However, the cardiovascular effects of *p*-synephrine have been opposed by several studies [98–101]. The study by Rodrigues et al. [102] was designed to investigate the herb-drug interactions between a standardized extract of *C. aurantium* and amiodarone, an antiarrhythmic medication, in rats. In the first step of the study, a single-dose of *C. aurantium* and amiodarone was administered orally to the rats at 164 mg/kg and 50 mg/kg doses, respectively. In the second step, the rats were pretreated with *C. aurantium* for 14 days, and on the 15th day, the amiodarone was administered at the same dose. On the other hand, the control group rats received vehicle only. The results demonstrated a significant enhancement of the peak plasma concentration of amiodarone in rats pretreated with *C. aurantium* extract, indicating a potential herb-drug interaction between *C. aurantium* extract and amiodarone [102]. Most recent clinical studies on 16 individuals have indicated that *C. aurantium* extract and its principal alkaloid, *p*-synephrine, had no adverse effects on the cardiovascular system after 15 days of treatment [103]. Moreover, Stohs et al. [67] reported approximately 30 human studies indicating that *p*-synephrine and bitter orange extracts do not result in cardiovascular effects; the effects in rodents cannot be directly extrapolated to humans at commonly used doses.

Regarding the adverse effects of formulations for body weight loss containing *Ephedra sinica* and *Citrus aurantium*, Arbo et al. used theuterotrophic assay to test the antiestrogenic activity of ephedrine, *p*-synephrine, *E. sinica*, and *C. aurantium* in immature female rats. Only ephedrine at 0.5 mg/kg/day presented a significative antiestrogenic effect [104].

## 5. Conclusion

In conclusion, studies regarding the bioactivity potential of *C. aurantium* have revealed that flowers, fruits, essential oils, and phytoconstituents of this plant exerted several biological effects including antimicrobial, antioxidant, cytotoxic, anxiolytic, antidiabetic, antiobesity, and anti-inflammatory activities. However, further long-term studies are needed in order to affirm its safety, especially regarding the plant-drug interactions and the proper dosage. *p*-Synephrine is a very poor adrenergic agonist and, therefore, is not expected to produce cardiovascular effects. It should be noted that it is banned by the NCAA which was assumed without evidence that it possesses cardiovascular risks and potential toxicity. It is not banned by the World Anti-Doping Agency (WADA) which deals with Olympic athletes.
